# Supplementary material for: A Quantitative Approach to Potency Testing for Chimeric Antigen Receptor-Encoding Lentiviral Vectors and Autologous CAR-T Cell Products, Using Flow Cytometry
Source: Pharmaceutics. 2025 Feb 25;17(3):303. doi: 10.3390/pharmaceutics17030303 (PMC11944512; doi:10.3390/pharmaceutics17030303)
Supplement: Supplementary file 1 [file pharmaceutics-17-00303-s001.zip › pharmaceutics-3467174-supplementary.pdf]

Supplementary material

Table S1. Characteristics of the CAR-T cell products included in the CD69-based potency assay test

Table S2. Summary of results obtained for CD69 expression and IFNγ levels in autologous CAR-T cell products

Figure S1. Gating strategy and antibody specificity of CD69-based potency assay

Figure S2. Development of CD69-based potency as a quality control-suitable assay

Figure S3. Development of CD69-based potency test for CARBCMA-LVV

Figure S4. CD19 expression levels in NALM6-CD19 low/medium/high expressing clones

Figure S5. Optimization of E:T ratio used in primary T-cell products (autologous CAR-T) cells

Figure S6. Effect of product's quality attributes on "CD69 fold activation" assay

Figure S7. Comparison of CD69 expression and IFNγ secreted levels

Table S1. Quality attributes of CAR-T cell products included in this study

CAR-T product	CD4/CD8 ratio	%CAR+ cells	CD69 fold activation
**ARI1_1**	0.69	29.2	4.19
**ARI1_2**	2.95	45.0	9.69
**ARI1_3**	1.16	57.5	5.78
**ARI1_4**	1.57	45.9	7.74
**ARI1_5**	0.93	41.3	7.47
**ARI1_6**	1.40	29.7	3.44
**ARI1_7**	2.09	24.8	3.62
**ARI1_8**	0.57	26.4	5.02
**ARI1_9**	1.55	44.8	4.95
**ARI1_10**	2.69	43.3	5.08

Table S2. Summary of results obtained for CD69 expression and IFNγ levels in autologous CAR-T cell products

Statistic	CD69 (MESF values)		IFNγ (pg/µl)	
E:T ratio	1:0 (Basal)	1:2 (Induced)	1:0 (Basal)	1:2 (Induced)
Mean	2979	13635	243.8	24265
SD	866.3	3144	242.6	19680
SEM	273.9	994.2	76.73	6223
CV	0.29	0.23	0.99	0.81
Median	3002	13219	171.6	16421
Minimum	1072	8553	4.84	3144
Maximum	4031	19249	677.4	57952
Range	2329	10696	672.6	54808

"SD" indicates Standard Deviation, "SEM" indicates Standard Error of the Mean", "CV" indicates "Coefficient of Variation"

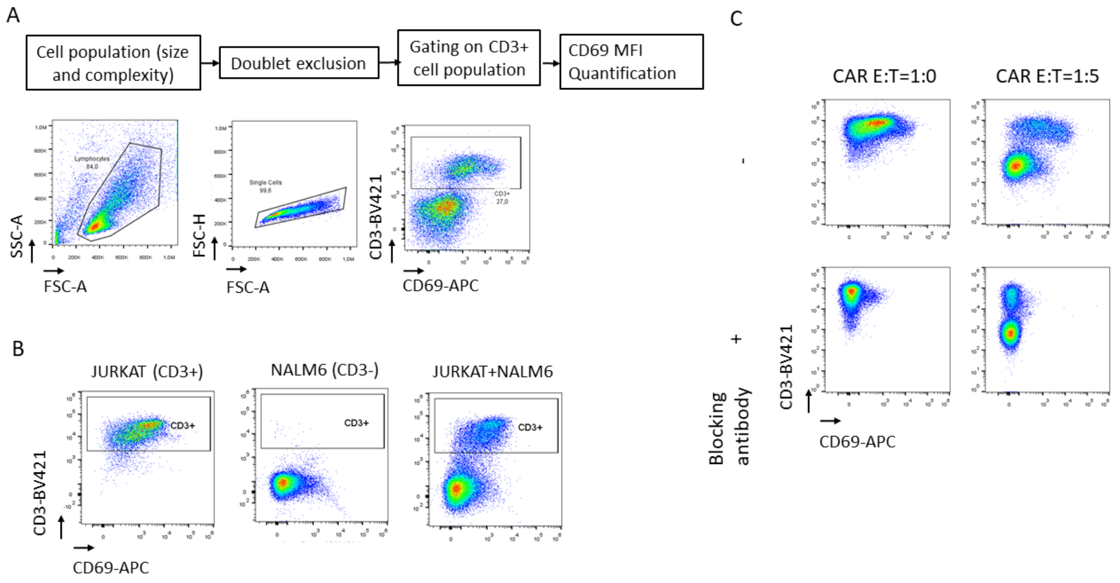

Figure S1. Gating strategy and antibody specificity of CD69-based potency assay. (a) Scheme and representative images of the gating-strategy used for the analysis of flow-cytometry data of CD69-based potency assay. **(b)** Specificity analysis of the anti-CD3 antibody used, using positive (Jurkat) and negative (NALM6) cell lines and discrimination of the two populations. **(c)** Specificity analysis of the anti-CD69 antibody used, using a blocking antibody against CD69 molecule.

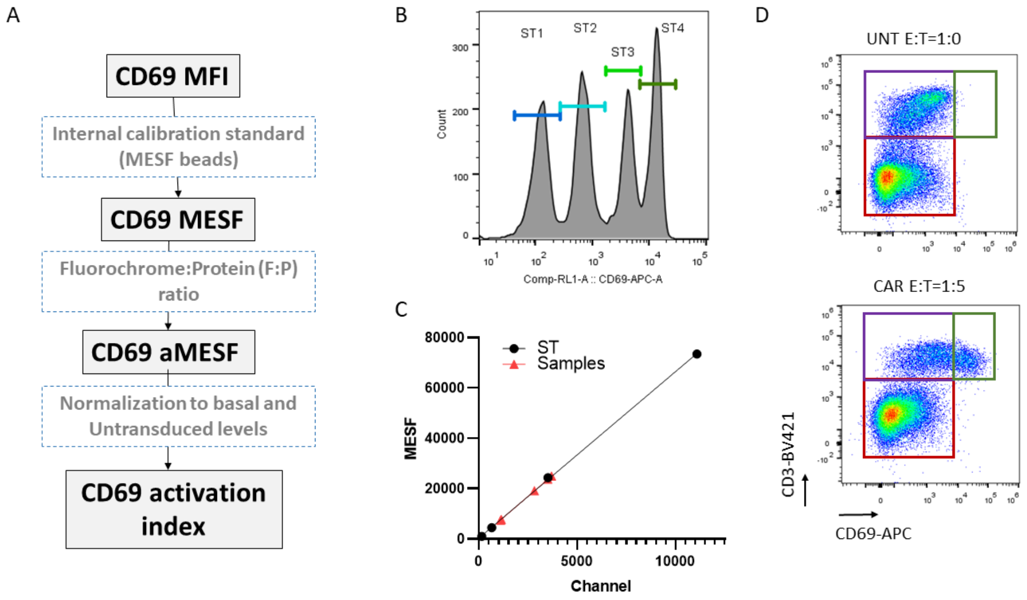

Figure S2. Development of CD69-based potency as a quality control-suitable assay. (a) Flow chart describing normalizations and data transformations to convert "CD69" MFI values to "CD69 activation index" **(b)** Representative image of fluorescence values obtained with MESF beads used as internal assay calibrator. **(c)** Standard curve (ST) obtained with MESF beads and typical values observed with assay's samples (within standard curve range). **(d)** Representative flow-cytometry images of CD69 activation assay. Upper panel corresponds to Untransduced T cells (UNT). Lower panel corresponds to CAR-expressing cells.

A

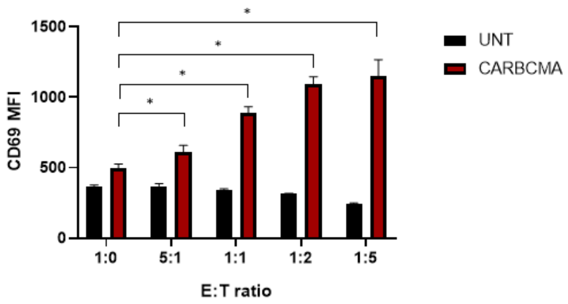

E:T ratio	1:0	5:1	1:1	1:2	1:5
CD69 Activation Index	1	1.21	1.93	2.56	3.51

B

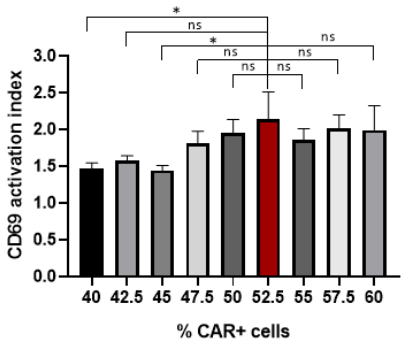

%CAR	40	42.5	45	47.5	50	52.5	55	57.5	60
CD69 Activation Index	1.47	1.58	1.44	1.81	1.96	2.14	1.86	2.01	1.99

Figure S3. Development of CD69-based potency test for CARBCMA-LVV. (a) CD69 MFI quantification in co-cultures of Untransduced and CARBCMA-expressing Jurkat cells and NALM6. Calculated CD69 activation index is shown in lower panel. **(b)** Impact of %CAR+ cells in CD69 activation index in 40-60% CAR+ interval. Calculated CD69 activation index is shown in lower panel. Mean of triplicates ±SD is shown. “n.s.” indicates no statistical significance. “*” indicates p≤0.05

A

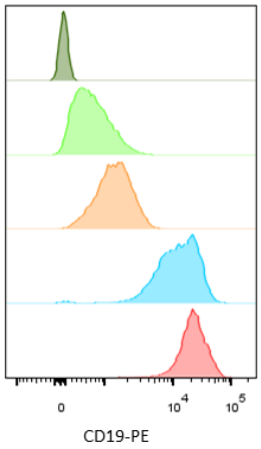

Sample	CD19 Molecules / cell
NALM6-CD19-KO	0
NALM6-CD19 cl.1	583
NALM6-CD19 cl.2	1317
NALM6-CD19 cl.4	12419
NALM6-WT	22692

Figure S4. CD19 expression levels in NALM6-CD19 low/medium/high expressing clones. (a) Representative histogram plots of flow-cytometry analysis of CD19 expression is shown in left panel. Quantification of CD19 molecules/cell is shown in left panel. NALM6-CD19 KO and NALM6 WT are also shown for reference.

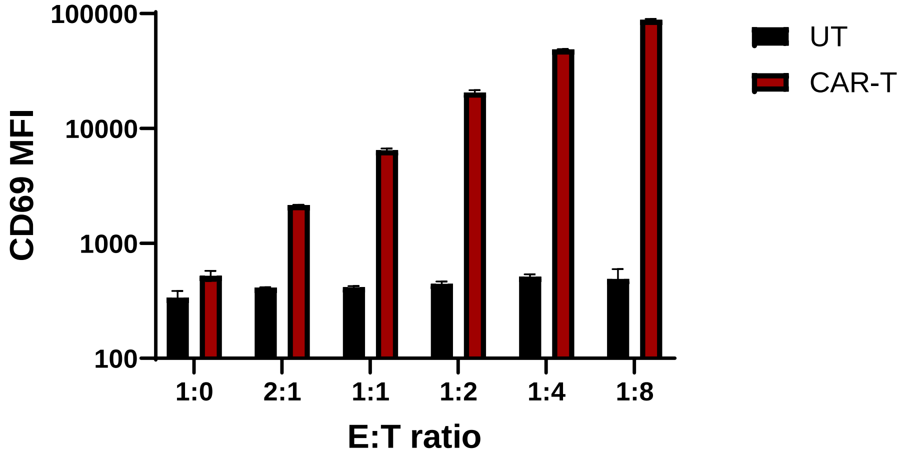

Figure S5. Optimization of E:T ratio used in primary T-cell products (autologous CAR-T) cells. CD69 MFI (shown in log10 scale) obtained when using different E:T ratios. Mean±SD of triplicates is shown.

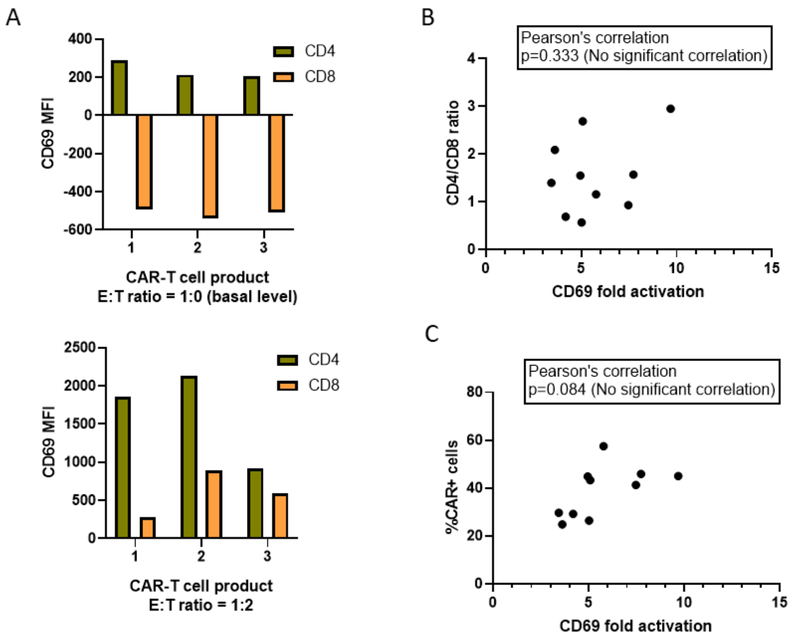

Figure S6. Effect of product's quality attributes on "CD69 fold activation" assay. (a) CD69 MFI in three different CAR-T cell products, in CD4+ and CD8+ cell population. Upper panel shows basal CD69 MFI. Lower panel shows CD69 MFI after 24h of co-culture with NALM6 cells at E:T ratio= 1:2. (b) Correlation between CD4/CD8 ratio and "CD69 fold activation". (c) Correlation between %CAR+ cells and "CD69 fold activation".

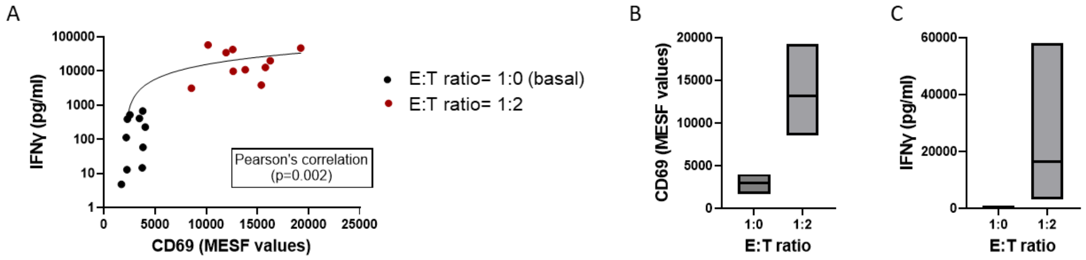

Figure S7. Comparison of CD69 expression and IFNγ secreted levels. (a) Correlation between IFNγ and CD69 expression levels in basal (E:T ratio=1:0) and induced conditions (E:T ratio=1:2). (b) Box plot showing variability and range of CD69 expression levels (MESF values). Line indicates the median. (c) Box plot showing variability and range of IFNγ levels. Line indicates the median.
